# Synthetic Cystic Fibrosis Sputum Medium Regulates Flagellar Biosynthesis through the *flhF* Gene in *Burkholderia cenocepacia*

**DOI:** 10.3389/fcimb.2016.00065

**Published:** 2016-06-14

**Authors:** Brijesh Kumar, Silvia T. Cardona

**Affiliations:** ^1^Department of Microbiology, University of ManitobaWinnipeg, MB, Canada; ^2^Department of Medical Microbiology and Infectious Diseases, University of ManitobaWinnipeg, MB, Canada

**Keywords:** SCFM, *Burkholderia cenocepacia*, cystic fibrosis, flagellum, motility, *flhF*

## Abstract

*Burkholderia cenocepacia* belongs to the *Burkholderia cepacia* complex (Bcc), a group of at least 18 distinct species that establish chronic infections in the lung of people with the genetic disease cystic fibrosis (CF). The sputum of CF patients is rich in amino acids and was previously shown to increase flagellar gene expression in *B. cenocepacia.* We examined flagellin expression and flagellar morphology of *B. cenocepacia* grown in synthetic cystic fibrosis sputum medium (SCFM) compared to minimal medium. We found that CF nutritional conditions induce increased motility and flagellin expression. Individual amino acids added at the same concentrations as found in SCFM also increased motility but not flagellin expression, suggesting a chemotactic effect of amino acids. Electron microscopy and flagella staining demonstrated that the increase in flagellin corresponds to a change in the number of flagella per cell. In minimal medium, the ratio of multiple: single: aflagellated cells was 2:3.5:4.5; while under SCFM conditions, the ratio was 7:2:1. We created a deletion mutant, Δ*flhF,* to study whether this putative GTPase regulates the flagellation pattern of *B. cenocepacia* K56-2 during growth in CF conditions. The Δ*flhF* mutant exhibited 80% aflagellated, 14% single and 6% multiple flagellated bacterial subpopulations. Moreover, the ratio of multiple to single flagella in WT and Δ*flhF* was 3.5 and 0.43, respectively in CF conditions. The observed differences suggest that FlhF positively regulates flagellin expression and the flagellation pattern in *B. cenocepacia* K56-2 during CF nutritional conditions.

## Introduction

*Burkholderia cenocepacia* is a species of the *Burkholderia cepacia* complex (Bcc), a group of at least 18 distinct species of extremely versatile gram-negative bacteria (Vandamme and Dawyndt, 2011). Ubiquitously present in the environment, Bcc bacteria have been found in various natural settings, such as soil, fresh water, and in association with plants, insects, and animals (Coenye and Vandamme, 2003; Vial et al., 2011). As opportunistic pathogens, Bcc bacteria are concerning because they establish chronic infections in the lung of people with the genetic disease cystic fibrosis (CF). Bcc infections in CF patients are characterized by a rapid decline in lung function and necrotizing pneumonia, which can result in early death (Isles et al., 1984). Eradication of Bcc infections is challenging due to the high antibiotic resistance of Bcc (Nzula et al., 2002; Conway et al., 2003; Sass et al., 2011). Therefore, it is important to identify new targets for therapeutic strategies to eliminate Bcc infections in CF and immunocompromised patients.

There is accumulating evidence that the nutritional environment of the infection site can modulate the virulence of infecting bacteria (Palmer et al., 2007). The sputum of CF patients is a complex mixture rich in mucous, amino acids, and carbohydrates that supports the proliferation of CF pathogens (Palmer et al., 2005, 2007). During growth in basal salt medium containing 12.5% CF sputum, *B. cenocepacia* clinical isolates showed differential regulation of several virulence factors, including antibiotic resistance, motility and iron uptake (Drevinek et al., 2008). With the goal of mimicking the nutritional conditions of the CF lung without the variability of CF sputum samples, a defined synthetic CF sputum medium (SCFM) was developed (Palmer et al., 2007) and utilized to analyse the transcriptomic response of *B. cenocepacia* growing in CF conditions (Yoder-Himes et al., 2010). Despite different platforms for data acquisition and analysis, the transcriptomes of *B. cenocepacia* grown with CF sputum or in SCFM showed that several genes putatively involved in flagellum biosynthesis were upregulated (Drevinek et al., 2008; Yoder-Himes et al., 2010).

Flagella are the locomotive organelles required for bacterial motility, chemotaxis, host colonization, biofilm formation, and dispersion of bacteria (Smith and Hoover, 2009). The flagellar structure, which is comprised of ~50 different proteins, extends outside of the cell surface. The outermost part, the flagellar filament is composed of flagellin (FliC) and is a major factor in inducing inflammatory responses (Eaves-Pyles et al., 2001). Although the flagellar structure is overall well conserved among bacteria, the location and number of flagella vary and are characteristic for flagellate bacterial species (Schuhmacher et al., 2015). Data on the flagellar morphology of *Burkholderia* species is scarce; two studies describe *B. pseudomallei* and *B. cenocepacia* as having one single polar flagellum, which contributes to virulence (Chua et al., 2003; Urban et al., 2004).

Although all Bcc species are capable of infecting CF patients, *B. cenocepacia* is one of the most prevalent species and it is associated with high morbidity and mortality (Zlosnik et al., 2014a,b). Despite the contribution of the flagellum to *B. cenocepacia* virulence and the existing evidence of increased flagellar gene expression in CF nutritional conditions, it is uncertain if upregulation of flagellar genes in *B. cenocepacia* results in flagellum structural or functional changes. In this study, we examined flagellin expression and flagellar morphology of *B. cenocepacia* grown in CF conditions compared to minimal medium. We found that the nutritional conditions of the CF lung induce expression of multiple flagella on the cell surface. We further demonstrate that the putative GTPase FlhF, which is involved in flagellar localization and biosynthesis in other species (Correa et al., 2005; Murray and Kazmierczak, 2006), is a positive regulator of motility, flagellin expression, and flagellar biosynthesis in *B. cenocepacia*.

## Materials and methods

### Bacterial strains, plasmids, and growth conditions

The bacterial strains and primers used in this study are summarized in Table 1 and Supplementary Table 1. The *B. cenocepacia* K56-2 wild type (WT) strain is a clinical isolate obtained from a CF patient. The bacterial strains were grown overnight in MOPS with glucose 5 or 20 mM, as indicated. Cells were washed in PBS and inoculated in SCFM, MOPS-glucose 20 mM or MOPS-glucose 5 mM with or without amino acids according to the design of the experiment. When appropriate, media was supplemented with trimethoprim (100 μg/ml for *B. cenocepacia* or 50 μg/ml for *Escherichia coli*), tetracycline (100 μg/ml for *B. cenocepacia* or 50 μg/ml for *E. coli*), kanamycin (25 μg/ml for *E. coli*) or xylose at 0.2% final concentration to induce the xylose inducible promoter on the p*flhF* plasmid. To construct p*flhF*, the *flhF* gene was PCR amplified (primers 1 and 2) and cloned into pAS2 backbone under a xylose inducible promoter. The resulting p*flhF* was introduced into WT and an unmarked *flhF* deletion mutant resulting in WT+p*flhF* and Δ*flhF*+p*flhF* strains, respectively. SCFM and MOPS-glucose 20 mM were prepared as described previously (Palmer et al., 2007). To study the effect of individual amino acids, MOPS-glucose 5 mM were supplemented with individual amino acids at the same concentration present in SCFM conditions. For growth assays, an aliquot of 200 μl growth media was inoculated with bacterial cells at a starting optical density of 600 nm (OD_600_) of 0.04 in triplicate in a 96-well plate. The plate was incubated at 37°C with continuous shaking for 24 h in a BioTek Synergy 2 plate reader. Readings were taken hourly at OD_600_ and values were converted to 1-cm-path-length OD_600_ by prior calibration with a GeneQuant™ III 4283, version 4283V1.6.

**Table 1:** **Strains and plasmids used in this study**.

**Strains/Plasmids**	**Features**	**References or Source**
***B. CENOCEPACIA***		
K56-2 (LMG18863)	Wild-type strain, ET12 clonal related to J2315, cystic fibrosis isolate	Mahenthiralingam et al., 2000; Duan et al., 2003
Δ*flhF*	Deletion of *flhF* in K56-2	This study
Δ*flhF* + p*flhF*	Complementation of Δ*flhF* strain with p*flhF* in trans	This study
K56-2 + p*flhF*	Overexpression of *flhF* in trans	This study
K56-2 + p*flhF*prom	*flhF* Operon Promoter::*luxCDABE* transcriptional fusion in pMS402	This study
K56-2 + pMS402	*lux*-based promoter reporter plasmid	Duan et al., 2003
***E. COLI***		
DH5α	F- φ80 *lacZΔM15 endA1 recA1 hsdR17*(rK^8^ mK^7^) *supE44 thi-1Δ gyrA96 (ΔlacZYAarg-F)U169 relA1*	Invitrogen
SY327	*araD* Δ(*lac pro*) *argE* (Am) *recA56* Rif^r^ *nalA λ pir*	Miller and Mekalanos, 1988
**PLASMIDS**		
pRK2013	Helper plasmid, Kan^r^	Figurski and Helinski, 1979
pGPI-SceI	DHFR, SceI recognition sequence, Tp^r^	Flannagan et al., 2008
pDAI-SceI	DHFR promoter controlling e-ISce-I, Tc^r^	Flannagan et al., 2008
pBK1	Fusion of upstream and downstream of *flhF* locus	This study
p*flhF*	*flhF* cloned under xylose inducible promoter in pAS2, Tp^*r*^	This study
p*flhF*prom	*flhF* Operon Promoter::*luxCDABE* transcriptional fusion in pMS402	This study
pMS402	Lux-based promoter reporter plasmid; Kan^r^, Tp^r^	Duan et al., 2003

Kan^r^, Kanamycin resistance; Tp^r^, Trimethoprim resistance; Tc^r^, Tetracycline resistance.

### Molecular biology techniques

The helper strain, *E. coli* pRK2013 (Table 1), was used for genetic manipulations in *B*. *cenocepacia* K56-2 via triparental mating. *E coli* SY327 Z-competent cells (Zyma Research, USA) were used to maintain the pBK1 plasmid. Polymerase chain reactions (PCR) were carried out with either *Taq* DNA polymerase (Qiagen) or HotStar HiFidelity *Taq* polymerase (Qiagen) with optimized conditions for each pair of primers. The DNA ligase and restriction enzymes (New England Biolabs) were used as recommended by manufacturers. QIAquick purification kit (Qiagen) and QIAprep Miniprep kit (Qiagen) were used to purify PCR products and plasmids respectively.

### Construction of *B. cenocepacia* K56-2 *flhF* unmarked deletion mutant

A previously developed unmarked deletion system for the genus *Burkholderia* was used to create an *flhF* deletion mutant with some modifications (Flannagan et al., 2008). Briefly, the upstream and downstream regions of the *flhF* locus (~480 bp) were synthesized by Blue Heron Biotechnology, USA, to form a fusion product with flanking XbaI and EcoRI restriction sites. The fusion product was digested with XbaI and EcoRI restriction enzymes and inserted into the XbaI-EcoRI digested pGPI-SceI. The resulting plasmid pBK1 was introduced into *B. cenocepacia* K56-2 WT by triparental mating, which lead to plasmid targeted insertion via homologous recombination. Conjugates with pBK1 integrated into the chromosome were selected on LB medium supplemented with trimethoprim. In the next step, pDAI-SceI, encoding genes for I-SceI endonuclease and tetracycline resistance, was introduced into the conjugate via triparental mating. Trimethoprim sensitive and tetracycline resistant clones were selected and screened for the *flhF* deletion by PCR analysis using primers flanking the *flhF* gene. The pDAI-SceI plasmid in positive clones was removed by sequentially sub-culturing the clones in antibiotic-free LB medium. The replica plate method was employed to confirm the loss of the plasmid indicated by tetracycline sensitivity. The presence of the deletion was also confirmed by PCR amplification (primers 3 and 4) and sequencing of the flanking regions of the *flhF* gene.

### Bioluminescence reporter assay for promoter activity

The PCR amplified *flhBAFG* operon promoter (primers 5 and 6) was fused to the *luxCDABE* reporter system (Aubert et al., 2013) in pMS402 resulting in the p*flhF*prom plasmid. Overnight cultures were washed in PBS and diluted to a starting OD of 0.04 in SCFM and MOPS-glucose 20 mM. Two hundred microliter aliquots were loaded in a black 96-well microplate with clear bottomed wells and incubated at 37°C with shaking. The OD_600_ and relative bioluminescence were measured using a Biotek Synergy 2 plate reader at 1-hour interval for 24 hrs.

### Western blot

The WT and mutant strains were grown overnight in SCFM, MOPS-glucose 20 mM and MOPS-glucose 5 mM with or without amino acids. To ensure equal amounts of protein were loaded, whole cell lysates were prepared from same volumes of cultures adjusted to an OD_600_ of 0.5. To prepare the whole cell lysates, the cells were thawed, pelleted and resuspended in 2X SDS loading dye. After boiling, the whole cell lysates were run in a 12% SDS-PAGE to separate protein and transferred onto PVDF membrane. Then, the FliC protein expression level was detected with the FliC specific primary anti-flagellin antibody raised in rabbit (kindly gifted by Dr. David Speert) and the secondary specific rabbit antibody tagged with alkaline phosphatase (Sigma-Aldrich, USA). Finally, the FliC-primary antibody complex and secondary antibody interaction was detected with a NBT/BCIP detection kit (Roche, USA). The ImageJ software was used to analyse the relative band intensity of the Western blot bands and the fold change was calculated in reference to MOPS-glucose.

### Motility assay, electron microscopy, and flagella staining

To examine WT and mutant swimming motility on semi-solid 0.3% agar, 5 μl of inoculum was stabbed on semi-solid plates. The plates were then incubated statically for 37°C for 24 h. The motility halos were recorded quantitatively by measuring the circular zone of turbidity, which corresponds to the bacteria swimming away from point of the inoculation. For electron microscopy sample preparation, a drop of the diluted overnight bacterial culture grown in SCFM and MOPS-glucose 20 mM was spotted on carbon-coated grids and stained with 2% uranyl acetate for 30 s. After drying the grids for 30 min, they were observed under the Hitachi H-7000 Electron Microscope. Flagella staining was performed as described previously (Heimbrook et al., 1989). Briefly, a sample of an overnight culture was taken with a loop and deposited on a clean microscope slide. The culture drop was covered with a cover slip and a few drops of Remel flagella stain (Thermo Scientific, USA) were applied at the end of cover slip. After 30 min, the stained flagella images were obtained with an AxioCamMR attached to an Axio ImagerZ1 (Carl Zeiss) at 100X magnification using a bright field filter.

### Statistical analysis

Motility halos of bacterial swimming and transcriptional activation of *flhF* operon promoter were compared and statistically analyzed. Data of two groups were analyzed with an unpaired Student's *t*-test, and one-way ANOVA followed by Dennett's multiple comparisons test was used for more than two groups. *P*-values were calculated using GraphPad Prism version 5.02 for Windows7, GraphPad Software, La Jolla California USA. Differences were considered significant when *P*-value was less than 0.05.

## Results

### CF nutritional conditions increase *B. cenocepacia* K56-2 swimming motility and flagellin expression

**Figure 1:**
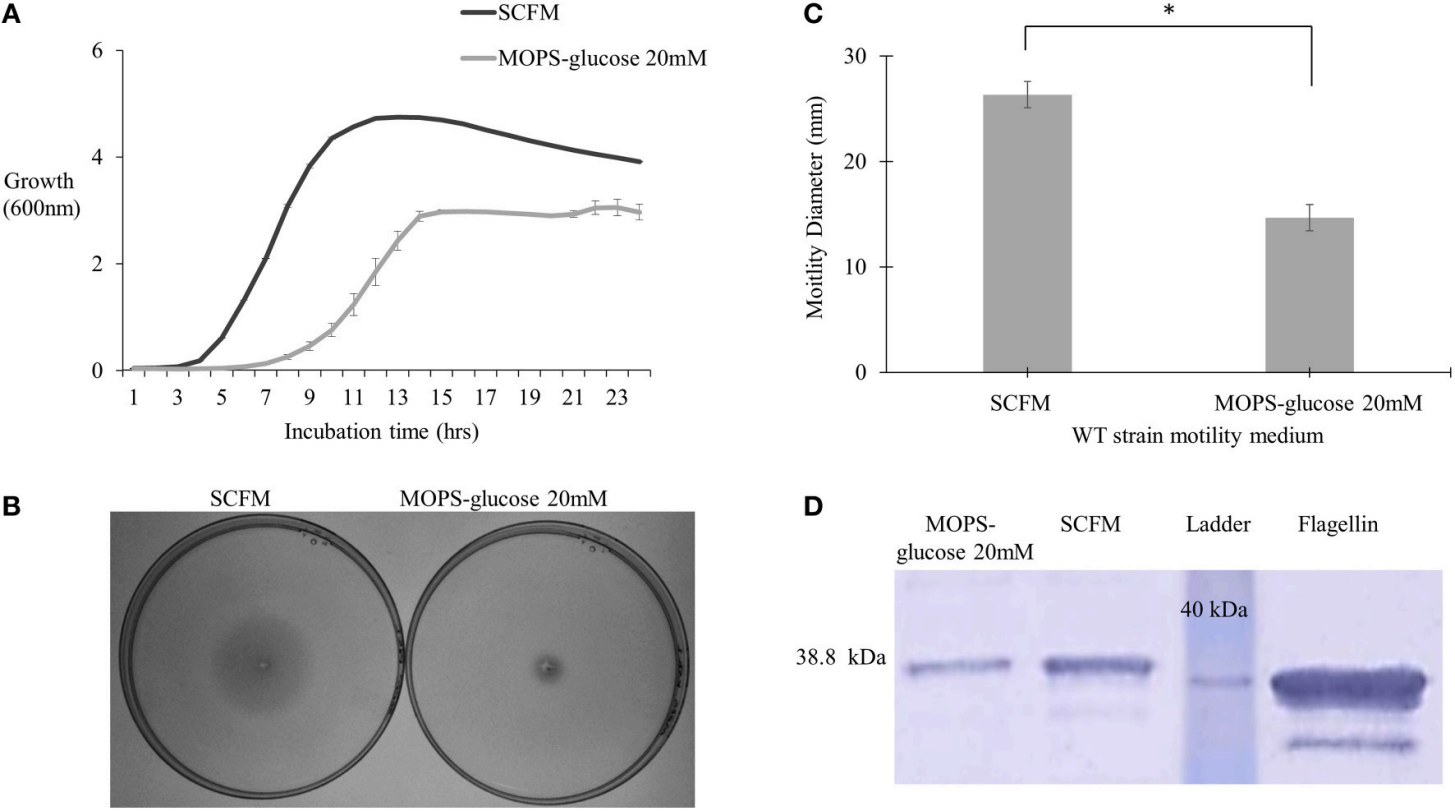
**Growth kinetics, motility phenotype and flagellin expression analysis of *B*. *cenocepacia* K56-2 WT. (A)** Growth kinetics of WT strain in SCFM and MOPS-glucose 20 mM. **(B,C)** Motility analysis. The motility of WT strain was examined in semi-solid SCFM and MOPS-glucose 20 mM 0.3% agar plates. Motility halos were recorded after 24 h of incubation time. Results correspond to three independent experiments and “*” denotes significant *p*-value (*p* < 0.05), which was calculated using Student's *t*-test. **(D)** Detection of flagellin by Western blot. Whole cell lysates and pure flagellin were run on 12% SDS –PAGE. The flagellin protein was detected using anti-flagellin primary antibody and secondary antibody cross-linked to alkaline phosphatase. Western blot and growth assays were performed twice independently. One representative experiment is shown.

To study regulation of virulence factors in *B. cenocepacia* K56-2 in CF nutritional conditions, we used SCFM, a medium developed from the average concentrations of nutrients found in CF sputum (Palmer et al., 2007). SCFM contains 3.2 mM glucose and a final total amino acid concentration of 19 mM. To compare SCFM-grown cells to those grown in minimal medium with an equivalent amount of energy source, MOPS-buffered minimal medium with 20 mM glucose was included as a control. Similar growth kinetics was observed between *P. aeruginosa* grown in CF sputum medium and MOPS medium containing 20 mM glucose (Palmer et al., 2005). Previous studies performed in the *B*. *cenocepacia* clinical isolates J2315 and AU1054, flagellar gene transcription was increased in response to CF nutritional conditions (Drevinek et al., 2008; Yoder-Himes et al., 2010). Since gene expression is subject to post-transcriptional regulation, we investigated whether the increase in flagellar gene expression resulted in elevated flagellin synthesis and/or motility. In agreement with previous results SCFM supported growth of *B. cenocepacia* K56-2 (WT) to a higher population density relative to MOPS-glucose 20 mM (Figure 1A). CF conditions also improved motility as the swimming ability of the WT strain increased by ~2-fold in semi-solid SCFM in comparison to MOPS-glucose 20 mM (Figures 1B,C). In addition, Western blot analysis of flagellin showed ~2.2-fold upregulation of flagellin in SCFM in comparison to MOPS-glucose 20 mM (Figure 1D). There was a slight difference between the migration distance of the native flagellin and the recombinant protein control produced in *E. coli*, likely due to the glycosylation of flagellin previously observed in *B. cenocepacia* (Hanuszkiewicz et al., 2014). Taken together, CF conditions induce flagellar gene upregulation, flagellin expression and swimming motility in *B*. *cenocepacia*.

### Individual amino acids increase *B. cenocepacia* K56-2 motility but not flagellin expression

**Figure 2:**
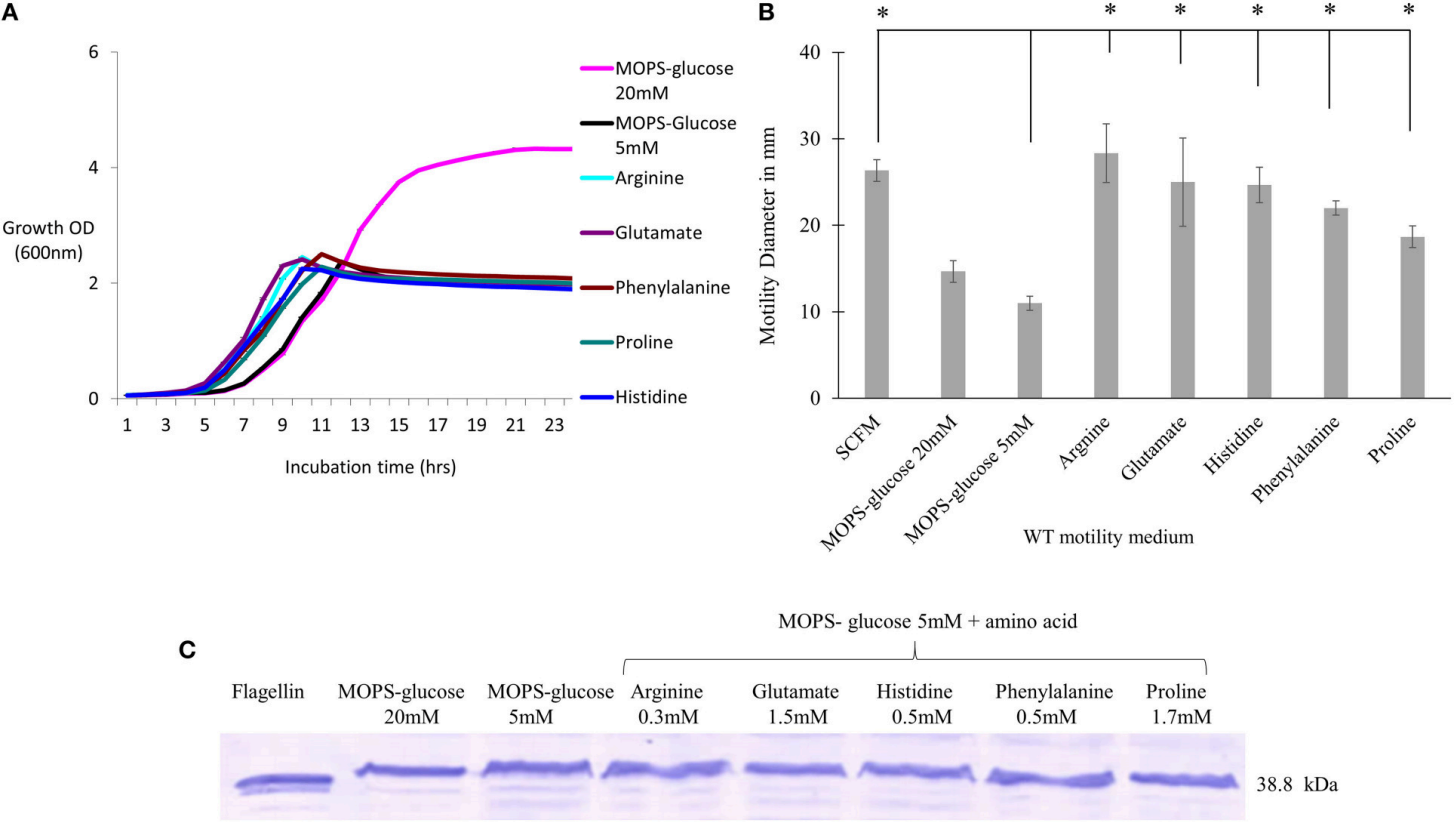
**The effect of individual amino acids effect on motility, growth and flagellin expression of *B*. *cenocepacia* K56-2 WT**. **(A)** Growth curves of WT strain in the presence of individual amino acids + MOPS-glucose 5 mM. **(B)** Motility halos of the WT strain in MOPS-glucose 5 mM + individual amino acid at the same concentration found in SCFM. The motility assay was performed three times independently and “*” denotes significant *p*-values (*p* < 0.05), obtained using One-Way ANOVA. **(C)** Western blot of WT whole cell lysates in the same condition as those used for the growth and motility analysis. Lane 1 shows the detection of purified flagellin protein as a positive control. Western blot and growth assays were performed twice independently. One representative experiment is shown.

We next investigated the contribution of the individual amino acids present in SCFM to the motility and flagellin expression phenotypes. We examined the growth and motility of the WT strain in MOPS with individual amino acids as a sole carbon source (Supplementary Table 2). Five amino acids, namely arginine, glutamate, histidine, phenylalanine and proline, were further investigated as they supported growth and induced high motility. To investigate the contribution of individual amino acids to the previously observed motility and flagellin phenotypes, cultures were grown with the aforementioned amino acids added individually at the same concentrations present in SCFM. As the concentration of each individual amino acid might be too low to support growth we added 5 mM glucose as the carbon source. Regardless of the presence of the particular amino acid, the cultures reached stationary phase at the same cell density as cultures grown in MOPS-glucose 5 mM only (Figure 2A). However, the individual amino acids significantly induced motility when compared to the MOPS-glucose 5 mM control (Figure 2B). This motility was comparable to what was observed in SCFM. To find out if individual amino acids induce increased flagellin expression, we performed a Western blot analysis of total proteins from cells grown in MOPS-glucose 5 mM supplemented with each of the five amino acids. Unlike what was observed for cells grown in CF conditions (Figure 1D), flagellin expression did not increase in the presence of individual amino acids (Figure 2C). Taken together these results suggest that the observed increase in motility associated with individual amino acids does not depend on increased flagellin expression and is more likely due to a chemotactic effect.

### CF conditions induce multiple flagella in *B. cenocepacia* K56-2

**Figure 3:**
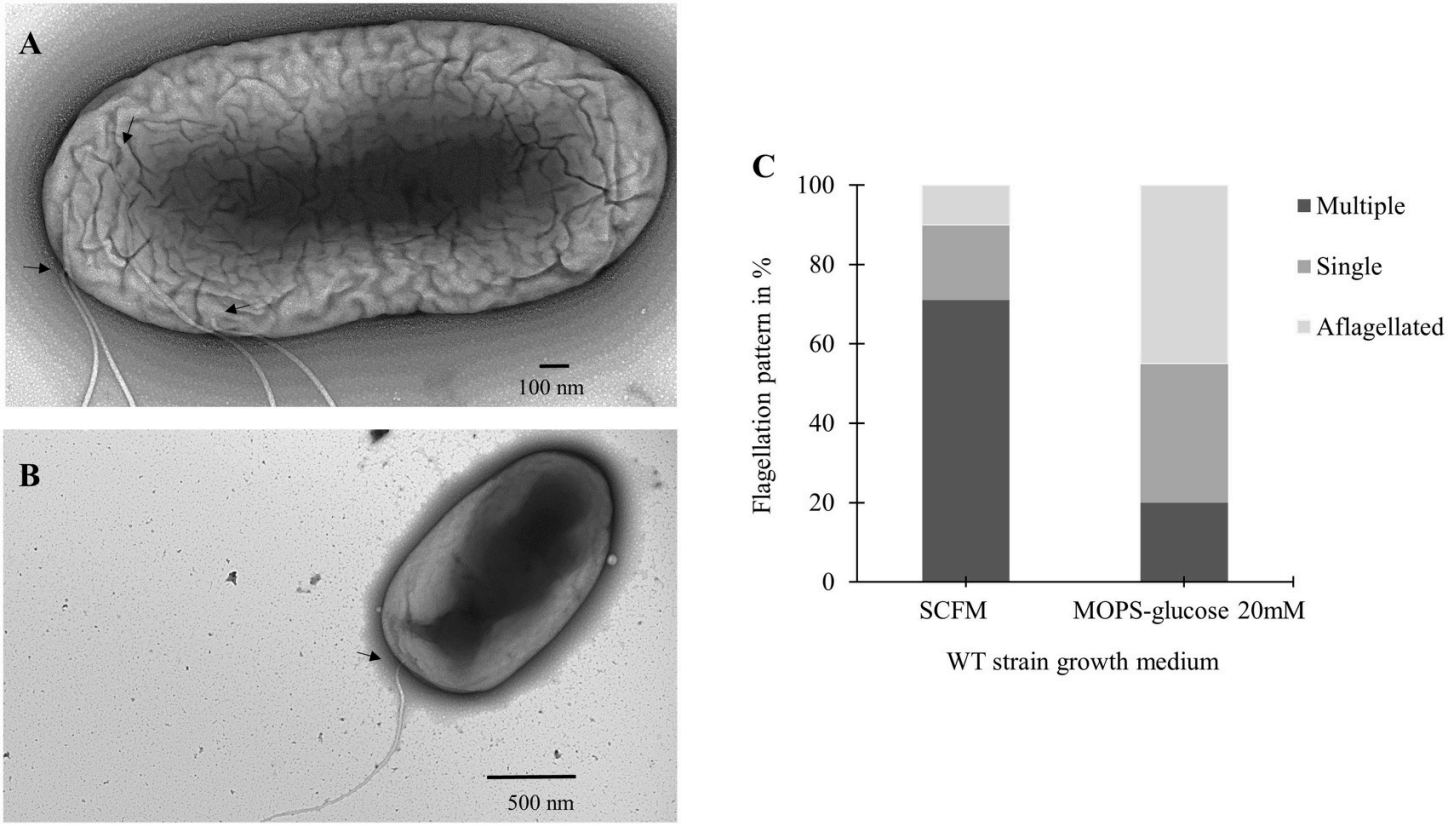
**Flagellar phenotypic analysis of *B*. *cenocepacia* K56-2 WT**. Transmission electron microscopy (TEM) of the WT strain in SCFM **(A)** and MOPS-glucose 20 mM **(B)**. **(C)** Distribution of the WT strain flagellation pattern in SCFM and MOPS-glucose after 24 h of growth. The arrows show the attachment site of flagella. Cell count was based on TEM images (*n* = 100).

To further investigate the increase in flagellin expression observed during growth in SCFM, we used transmission electron microscopy (TEM) and the flagella stain technique (Heimbrook et al., 1989). Previous work has shown that *B. cenocepacia* cells grown on an LB plate are monotrichous (Urban et al., 2004). Similarly, TEM images showed cells grown in MOPS-glucose 20 mM as having a single, long polar flagellum (Figure 3B). However, in TEM images of cells grown in SCFM, an increased number of flagella was observed (Figure 3A). Some flagella were detached from the cells and were broken highlighting the previously described fragility of *B. cenocepacia* flagella (Urban et al., 2004). The microscopic images suggested that the increase in *B. cenocepacia* flagellin protein expression and motility are likely due to multiple flagellated bacterial subpopulations present in SCFM condition, which are more numerous than in MOPS-glucose 20 mM. To quantify the differences in the flagellation patterns between the two conditions, we classified 100 cells shown in the images, as having multiple flagella, a single flagellum, or being aflagellated. The ratio of multiple: single: aflagellated cells under SCFM conditions was 7:2:1, while in MOPS-glucose 20 mM conditions, the ratio was 2:3.5:4.5 (Figure 3C). In SCFM-grown cells, flagella were localized laterally but close to the pole (Figure 3B and Supplementary Figure 1); however, in MOPS-glucose 20 mM, flagella were mostly localized at the pole (Figure 3A and Supplementary Figure 1). Also, 45% of the bacteria did not have flagella in MOPS-glucose 20 mM, while in SCFM only 10% of bacteria were aflagellated (Figure 3C). To confirm the flagellation phenotype in SCFM and MOPS-glucose 20 mM, we stained *B. cenocepacia* K56-2 flagella with the Remel flagella stain dye and the stained flagella were observed under bright-field microscopy. In SCFM, the WT strain displayed multiple flagella localized close to the pole. On the other hand, MOPS-glucose 20 mM grown cells demonstrated a single polar flagellar morphology, similar to what was observed with TEM (Supplementary Figure 2).

### *flhF* regulates flagellin expression and flagellation pattern

**Figure 4:**
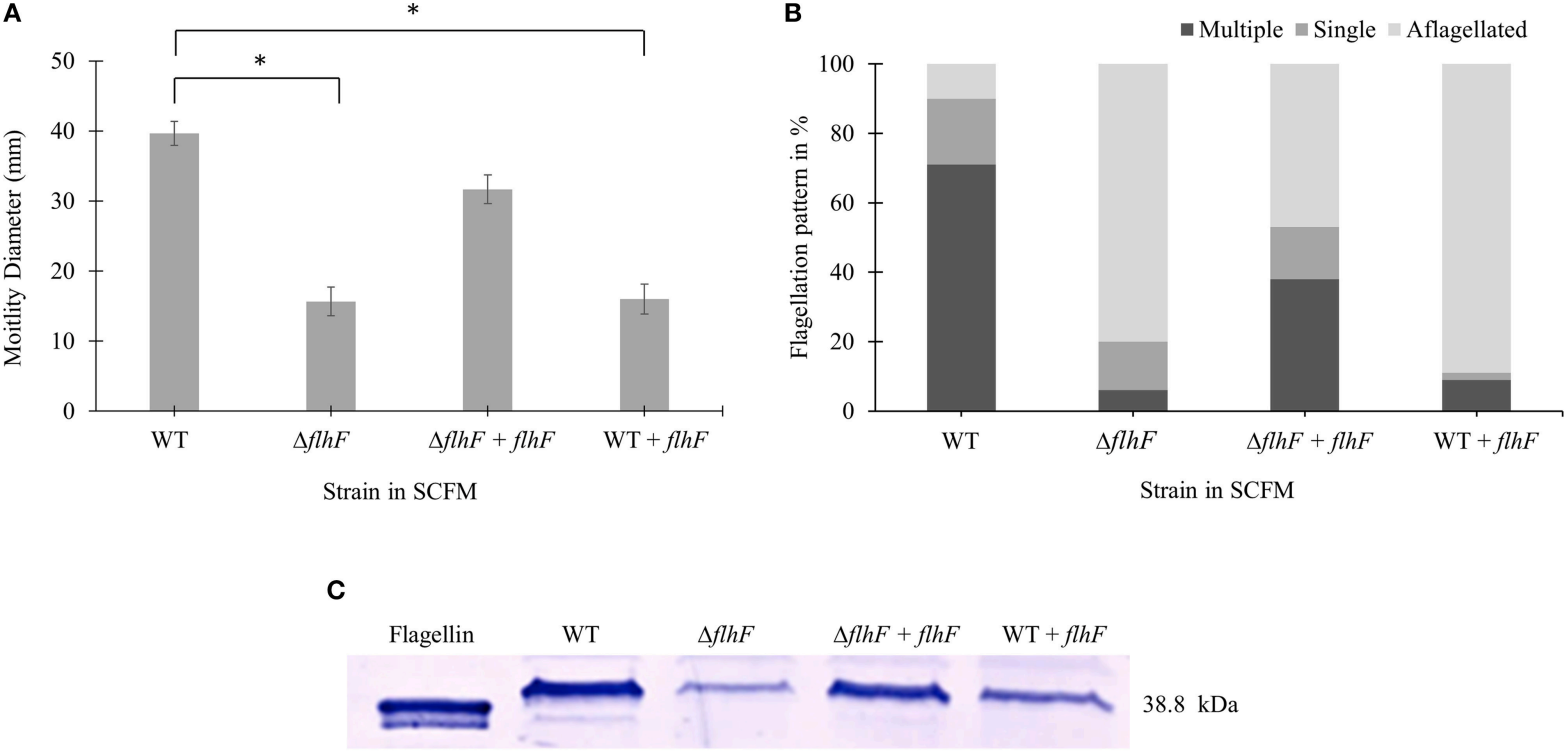
**Motility, flagellar morphology and flagellin expression analysis of *flhF* deletion and overexpression in strains of *B. cenocepacia* K56-2 WT**. Motility **(A)**, flagellation pattern **(B)** and flagellin expression levels **(C)** of WT, Δ*flhF* mutant, Δ*flhF*+*flhF complement* and WT+*flhF* overexpression strain. The motility assay was performed three times independently. One-Way ANOVA was performed to obtain *p*-values and “*” denotes significant *p*-values (*p* < 0.05). The WT motility was used as control for comparisons.

*flhF* encodes a signal recognition type (SRP)-type GTPase protein, which is one of the regulators of the hierarchy for flagella biosynthesis (Carpenter et al., 1992; Bange et al., 2011). Previous studies showed a role of *flhF* in flagellar biosynthesis and localization in various bacterial genera such as *Pseudomonas, Vibrio, Campylobacter*, and *Shewanella* (Correa et al., 2005; Murray and Kazmierczak, 2006; Kusumoto et al., 2008; Balaban et al., 2009; Schuhmacher et al., 2015). We then investigated if the *flhF* gene of *B. cenocepacia* K56-2 strain was involved in regulating the number and localization of flagella during growth in SCFM. We constructed *flhF* unmarked deletion mutant (Δ*flhF)* and characterized its motility, flagellin expression and flagellar morphology in CF conditions. The Δ*flhF* mutant exhibited 2.5-fold reduction in motility and ~4-fold down-regulation in flagellin expression (Figures 4A,C). When Δ*flhF* was complemented with the *flhF* gene *in trans* under a xylose inducible promoter, both motility and flagellin expression were restored although not to WT levels. Further, we examined the effect of FlhF protein overexpression in the WT strain. Overexpression of FlhF caused 2.4 and ~2.3-fold reduction in motility and flagellin expression, respectively in the WT strain (Figures 4A,C). To study whether the *flhF* gene regulates the flagellation pattern of *B. cenocepacia* K56-2 during growth in CF conditions, we obtained TEM images of WT and Δ*flhF* grown in SCFM. The Δ*flhF* mutant exhibited 80% aflagellated, 14% single and 6% multiple flagellated bacterial subpopulations (Figure 4B). This indicates that Δ*flhF* has a major role in the biosynthesis of flagella. The ratio of multiple to single flagella in WT and Δ*flhF* were 3.5 and 0.43 respectively (Figure 4B). When Δ*flhF* was complemented (Δ*flhF*+p*flhF*) the flagellation pattern was restored although not completely to the WT level (Figure 4B). These observations suggest that the *flhF* gene is also involved in the flagellation pattern observed in CF conditions. Noticeably, in Δ*flhF* and WT overexpressing *flhF*, large bacterial subpopulations exhibited aflagellated morphology (Figure 4B) indicating that perturbations of the FlhF protein levels dramatically alter the proportion of flagellated cells. Taken together these results suggest that FlhF positively regulates flagellin expression and flagellation pattern in *B. cenocepacia* K56-2.

### The *flhBAFG* operon is induced in CF conditions

**Figure 5:**
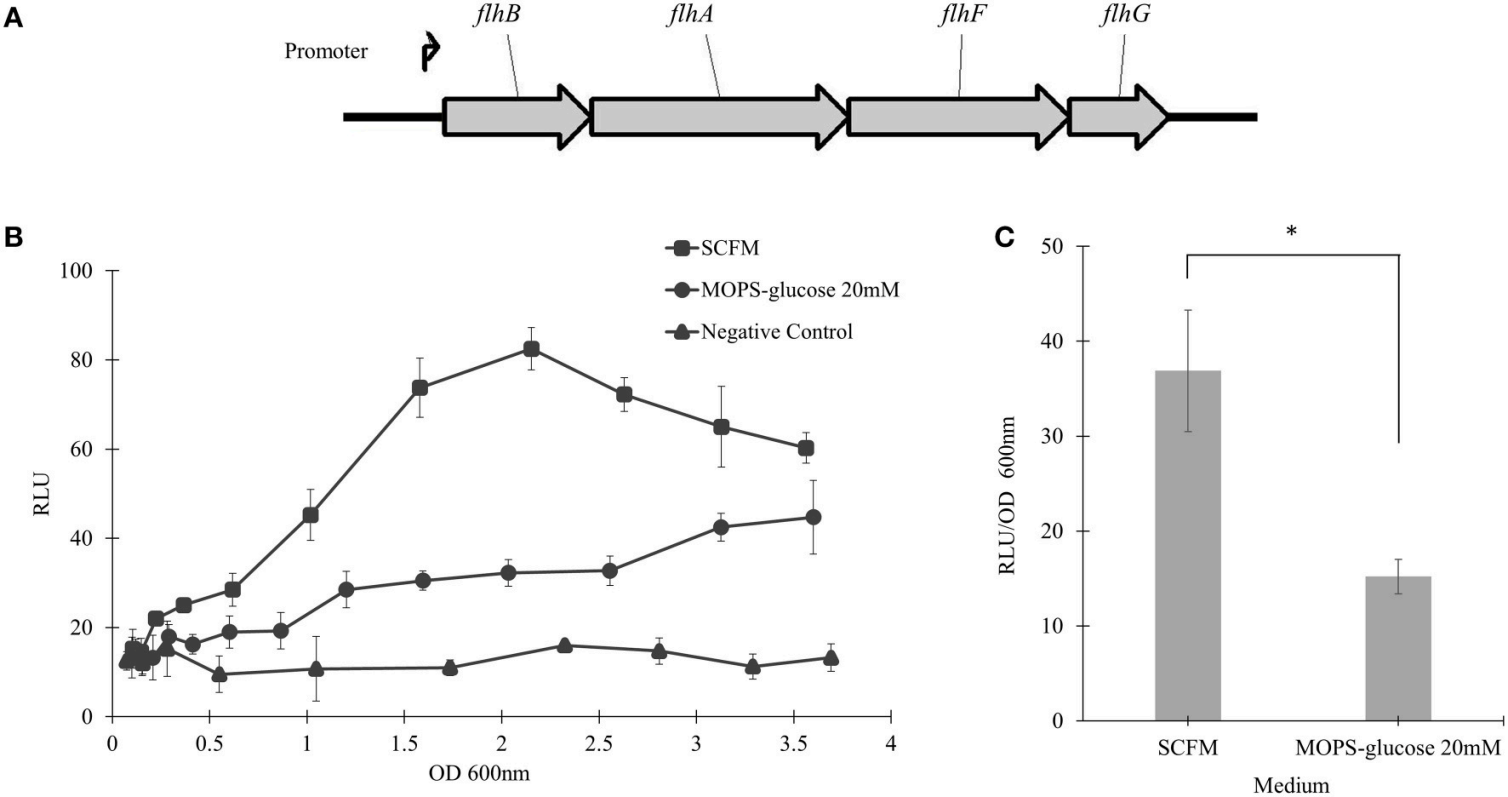
**The *flhBAFG* operon and its transcription activity. (A)** Schematic of the flagellar *flhBAFG* operon. The angle arrow represents the promoter upstream of the *flhB* gene. To examine transcriptional activity, a 531 bp fragment containing the *flhB* promoter was amplified and cloned upstream of the lux operon in the bioreporter system. **(B)** The *flhF* promoter sequence was transcriptionally fused upstream to *luxCDABE* gene cassette (*flhF-lux*) on a plasmid (p*flhF*prom) and introduced into the WT strain. Bioluminescence was measured hourly in response to activation of the *flhF* operon promoter in SCFM and MOPS-glucose 20 mM conditions. The transcriptional activity assay was performed three times showing similar results. One representative graph is shown. **(C)** The graph shows RLU/OD_600_ at the mid-exponential phase of OD_600_ 2.2 in SCFM and MOPS-glucose 20 mM. “*” denotes significant *p*-value (*p* < 0.05), which was calculated using Student's *t*-test.

To examine the transcriptional activity of *B. cenocepacia* WT *flhF* operon, the WT strain was transformed with a plasmid encoding the *flhBAFG* operon promoter (Figure 5A), upstream to the *luxCDABE* genes cluster in a transcriptional reporter system. The WT strain harboring the reporter system was grown in SCFM or MOPS-glucose 20 mM, and the bioluminescence produced upon transcriptional activation was plotted against the OD_600_ of bacterial growth. In SCFM, the bioluminescence reached a maximum at an approximate OD_600_ of 2.2, which corresponds to mid-exponential growth (Figure 1A) and the signal was ~2.6-folder higher in SCFM than in MOPS-glucose 20 mM (Figure 5B). Statistical analysis of the differences between relative luminescence in SCFM and control media during the mid-exponential phase showed significant increase in the activation of the *flhF* operon promoter in SCFM (Figure 5C). Overall, these results suggest that CF conditions increase transcription of the *flhBAFG* operon and that the increased transcription of the *flhF* gene has an effect on flagellin expression and flagellation pattern, which in turns increases motility.

## Discussion

The aim of this study was to investigate *B. cenocepacia* motility and regulation of the virulence factor flagellin in SCFM, which mimics CF nutritional conditions. SCFM supported growth to high population densities (Figure 1A), which is in agreement with the growth of *B. cenocepacia* J2315, HI2424 and *Pseudomonas aeruginosa* in similar conditions (Palmer et al., 2007; Yoder-Himes et al., 2009, 2010). The motility of *B. cenocepacia* K56-2 and expression of flagellin increased in SCFM in comparison with minimal medium (Figures 1B–D). Motility, but not expression of flagellin, was also increased in response to five amino acids present in SCFM (Figure 2). These amino acids were arginine, glutamate, histidine, phenylalanine, and proline all of which *B. cenocepacia* could utilize as a sole carbon source (Supplementary Table 2). Studies have shown that amino acids act as chemoattractants in bacterial species such as *E. coli* and *Bacillus subtilis* (Yang et al., 2015). Bacteria move toward preferential nutrient sources as demonstrated by the strong correlation between chemotaxis and amino acids utilization in *E. coli* (Yang et al., 2015). As we did not find an increase in flagellin expression levels in cells grown in MOPS-glucose 5 mM supplemented with individual amino acids in comparison to cells grown in glucose MOPS-glucose 5 mM, we conclude that amino acids increase motility through a chemotactic effect in *B. cenocepacia* that is independent of increased flagellar synthesis (Figure 2C).

Electron microscopy images of cells grown in CF nutritional conditions showed cells with multiple polar or laterally localized flagella. Also, the total flagellated subpopulations of cells grown in SCFM were larger than the ones grown in MOPS-glucose 20 mM (Figure 3). Flagellum biogenesis is a very complex process where dozens of structural and regulatory genes are expressed hierarchically (Tsang and Hoover, 2014). Bacterial species are typically described as presenting polar or lateral flagellar morphology. However, under certain conditions bacteria can adjust their flagellation patterns. Lateral flagella are observed in viscous environments, whereas polar flagella are noticeable in swimming conditions (Atsumi et al., 1996; McCarter, 2004). In *V. parahaemolyticus*, an iron-depleted growth medium was shown as a second signal, after viscosity, to induce lateral flagella (McCarter and Silverman, 1989). Similarly, our results suggest that CF nutritional conditions can mediate a change in flagellar number and localization (Figure 3).

Although the process of flagellar biogenesis is conserved in most bacterial species, the regulatory mechanisms vary (Jang et al., 2014). In *Vibrio, Campylobacter, Shewanella,* and *Pseudomonas* species, FlhF is a regulatory protein associated with positioning and biosynthesis of flagella (Murray and Kazmierczak, 2006; Kusumoto et al., 2008; Balaban et al., 2009; Schuhmacher et al., 2015). The *flhF* gene is present in an operon, *flhBAFG*, in *B. cenocepacia* K56-2 chromosome 1 (Winsor et al., 2008). Upon deleting *flhF*, we found that flagellin expression and motility was reduced (Figure 4). The aflagellated morphology of the Δ*flhF* mutant indicates a primary role of *flhF* in flagellar synthesis (Figure 4B). However, the *flhF* gene is also involved in flagellar localization as indicated by the ratio of multiple to single polar flagella in WT strain Δ*flhF* mutant. Overexpression of FlhF in *Pseudomonas* and *Vibrio* species resulted in non-polar or polar hyperflagellation, respectively depending on the species (Kazmierczak and Hendrixson, 2013). On the contrary, *B*. *cenocepacia* FlhF overexpression caused reduction in flagellin, motility and flagella biosynthesis (Figure 4). This reduction suggests that after a certain amount of FlhF protein is produced, it may negatively regulate its own gene expression and flagellar biosynthesis. Further, the transcriptional reporter system identified that the *flhF* operon was more activated in SCFM than in MOPS-glucose 20 mM (Figure 5).

On the basis of our results, it is likely that flagellin and flagellar morphology regulation may play a role in the initial stages of colonization when the nutritional environment of the CF lung is sensed by *B. cenocepacia.* The increase in motility may contribute to spreading and development of *B. cenocepacia* chronic infection. The role of bacterial flagella in the pathogenicity of *B. cenocepacia* has been intensively studied due to various reasons. Flagellum acts as an adhesin to contact epithelial cells during the initial stages of colonization (Mahenthiralingam et al., 2005) and helps in invasion of host cells (Tomich et al., 2002). Flagellin is sensed as a ligand of Toll-like receptor 5, which results in induction of the inflammatory host defense response for pathogen eradication (Hayashi et al., 2001). Since various studies have already demonstrated the role of flagella as a virulence factor (Tomich et al., 2002; Urban et al., 2004; Balloy et al., 2007; Roberts et al., 2015), regulatory proteins that inhibit flagellar biosynthesis can be potential targets to develop therapeutic molecules to treat *B. cenocepacia* infections.

## Author contributions

BK designed and performed the experiments, interpreted the data and wrote the manuscript. SC conceived the research approach, contributed to writing and edited the final version of the manuscript.

### Conflict of interest statement

The authors declare that the research was conducted in the absence of any commercial or financial relationships that could be construed as a potential conflict of interest.

## Supplementary material

Supplementary Table 1 **Primers used in this study**.

Supplementary Table 2 **Individual amino acids as a sole carbon source: Growth and motility of the *B. cenocepacia* K56-2 WT was examined in MOPS containing 5 mM amino acids as individual carbon sources**. The symbols “++,” “+,” and “−” represent high, moderate and no growth or motility respectively.

Supplementary Figure 1 **TEM images of *B. cenocepacia* K56-2 WT. (A,B)** Multiple flagella present on WT strain cell surface in CF nutritional conditions. **(C,D)** Single and multiple polar flagella in MOPS-glucose 20 mM.

Supplementary Figure 2 **Bright-field microscopic images of *B. cenocepacia* K56-2 WT stained flagella: The bacterial flagella were stained using Remel flagella dye. (A)** Stained multiple flagellated WT strain cells in CF nutritional conditions. **(B)** Stained polar flagellum or aflagellated cells in MOPS-glucose 20 mM. Scale bars represent 2 μm in all images.
