# Supplementary material for: Synthetic Cystic Fibrosis Sputum Medium Regulates Flagellar Biosynthesis through the flhF Gene in Burkholderia cenocepacia
Source: Front Cell Infect Microbiol. 2016 Jun 14;6:65. doi: 10.3389/fcimb.2016.00065 (PMC4905959; doi:10.3389/fcimb.2016.00065)
Supplement: Supplementary Table 1 — Primers used in this study. [file Table1.DOCX]

**Supplementary Table 1.**

| 1. *flhF* Forward | ATAGCATATGTTGAACATTCGCAAATTCACCG |
| --- | --- |
| 1. *flhF* Reverse | ATATTCTAGATTATCCAAATCGCACCTCGTGCA |
| 1. ∆*flhF* confirm Forward | GCAAGCGATGCTGCGTCAACAGAA |
| 1. ∆*flhF* confirm Reverse | AAGATTCACCACGGTGGACGTGCA |
| 1. *flhF*prom Forward | TAATCTCGAGGGTGATGACGATGAAGAAGCTCG |
| 1. *flhF*prom Reverse | ATATGGATCCTGGCGGCTTCAGGTCTTGTCGA |

* Bases underlined represent restriction enzyme recognition sites.
