# Supplementary material for: Synthetic Cystic Fibrosis Sputum Medium Regulates Flagellar Biosynthesis through the flhF Gene in Burkholderia cenocepacia
Source: Front Cell Infect Microbiol. 2016 Jun 14;6:65. doi: 10.3389/fcimb.2016.00065 (PMC4905959; doi:10.3389/fcimb.2016.00065)
Supplement: Supplementary Table 2 — Individual amino acids as a sole carbon source: Growth and motility of the B. cenocepacia K56-2 WT was examined in MOPS containing 5 mM amino acids as individual carbon sources. The symbols “++,” “+,” and “−” represent high, moderate and no growth or motility respectively. [file Table2.DOCX]

**Supplementary Table 2.**

| **Amino Acid** | **Growth** | **Motility** |
| --- | --- | --- |
| Alanine | ++ | + |
| Arginine | ++ | ++ |
| Aspartate | + | + |
| Cysteine | - | - |
| Glutamate | + | ++ |
| Glycine | - | - |
| Histidine | + | ++ |
| Isoleucine | - | - |
| Leucine | - | - |
| Lysine | + | + |
| Methionine | - | - |
| Ornithine | - | - |
| Phenylalanine | ++ | ++ |
| Proline | + | ++ |
| Serine | - | - |
| Threonine | - | - |
| Tyrosine | - | - |
| Tryptophan | + | + |
| Valine | - | - |
