# Supplementary figures and images for: Synthetic Cystic Fibrosis Sputum Medium Regulates Flagellar Biosynthesis through the flhF Gene in Burkholderia cenocepacia
Source: Front Cell Infect Microbiol. 2016 Jun 14;6:65. doi: 10.3389/fcimb.2016.00065 (PMC4905959; doi:10.3389/fcimb.2016.00065)

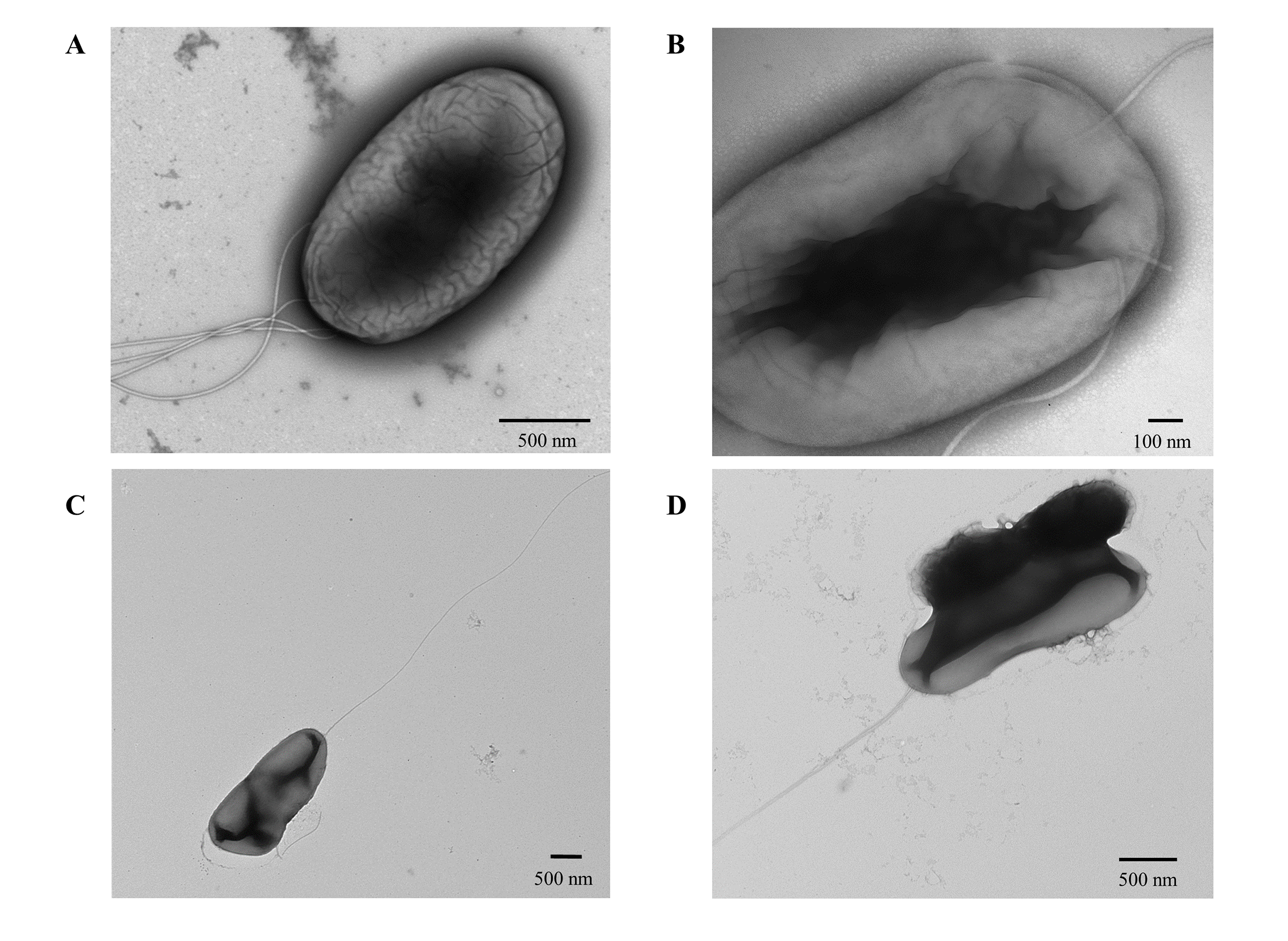

Supplement: Supplementary Figure 1 — TEM images of B. cenocepacia K56-2 WT. (A,B) Multiple flagella present on WT strain cell surface in CF nutritional conditions. (C,D) Single and multiple polar flagella in MOPS-glucose 20 mM. [file Image1.TIF]

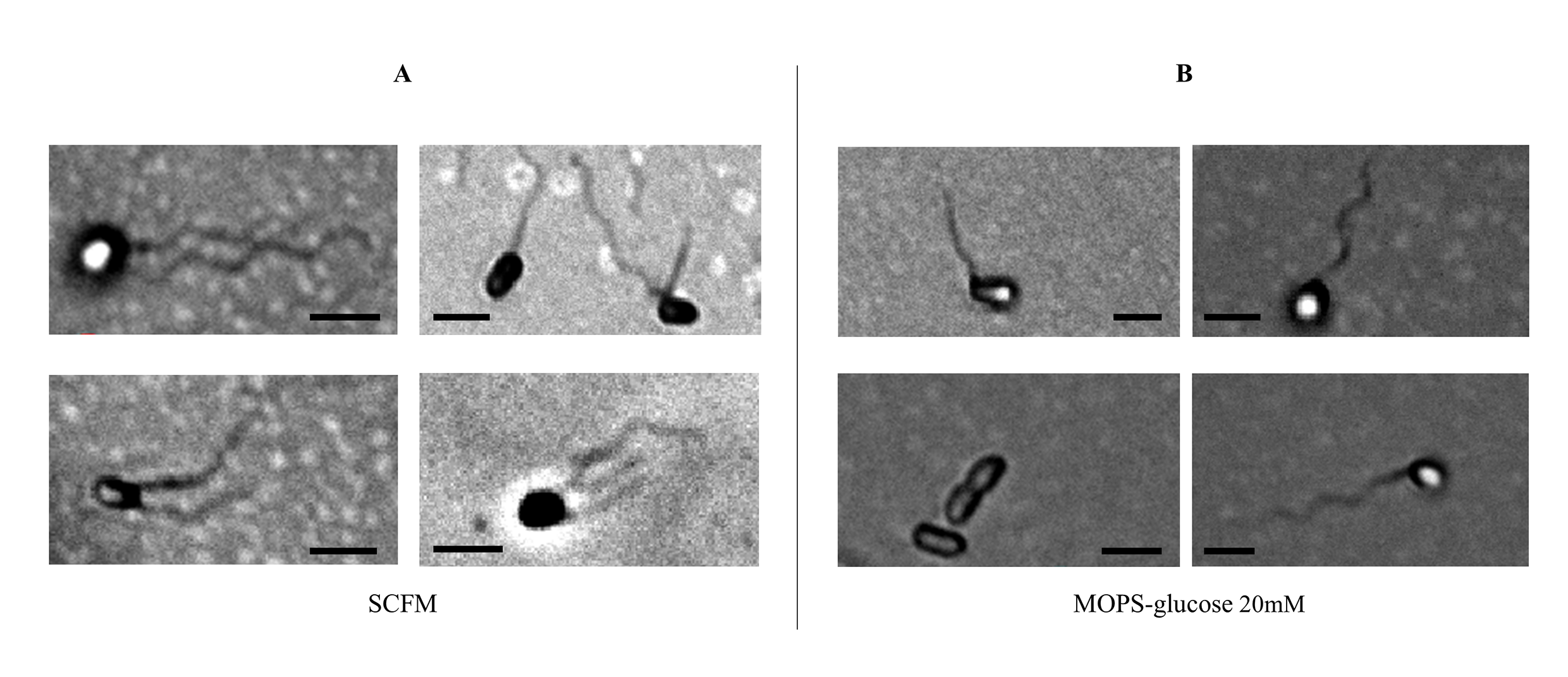

Supplement: Supplementary Figure 2 — Bright-field microscopic images of B. cenocepacia K56-2 WT stained flagella: The bacterial flagella were stained using Remel flagella dye. (A) Stained multiple flagellated WT strain cells in CF nutritional conditions. (B) Stained polar flagellum or aflagellated cells in MOPS-glucose 20 mM. Scale bars represent 2 μm in all images. [file Image2.TIF]
